# The importance of mechanosensitive cell mediated prostaglandin and nitric oxide synthesis in the pathogenesis of apical periodontitis: comparative with chronic periodontitis

**DOI:** 10.1007/s00784-024-05721-3

**Published:** 2024-05-25

**Authors:** Fatih Ozcelik, Seyda Ersahan, Dursun Ali Sirin, Ilbey Kayra Ozçelik, Yelda Erdem Hepsenoglu, Burak Karip

**Affiliations:** 1 Department of Medical Biochemistry, Sisli Hamidiye Etfal Training and Research Hospital, University of Health Sciences Turkiye, Istanbul, Türkiye; 2 Department of Endodontics, Faculty of Dentistry, Istanbul Medipol University, Istanbul, Türkiye; 3 Department of Endodontics, Faculty of Dentistry, University of Health Sciences, Istanbul, Türkiye; 4 Faculty of Medicine, Istinye University, Istanbul, Türkiye; 5 Department of Anatomy (Dentist), Hamidiye Faculty of Medicine, University of Health Sciences Turkiye, Istanbul, Türkiye

**Keywords:** Gingival crevicular fluid, Apical periodontitis, Chronic periodontitis, Prostaglandin E2, Nitric oxide, IL-10

## Abstract

**Objectives:**

Mechano-sensitive odontoblast cells, which sense mechanical loading and various stresses in the tooth structure, synthesize early signaling molecules such as prostaglandin E2 (PGE2) and nitric oxide (NO) as an adaptive response. It is thought that these synthesized molecules can be used for the diagnosis and treatment of periodontal and periapical diseases. The aim of this study was to investigate the relationship between the severity of apical periodontitis (AP) and chronic periodontitis (CP) and serum (s) TNF-α, IL-10, PGE2 and NO levels, as well as PGE2 and NO levels in gingival crevicular fluid (GCF) samples.

**Materials & methods:**

A total of 185 subjects were divided into three categories: AP group (*n* = 85), CP group (*n* = 50) and healthy control group (*n* = 50). The AP group was divided into 3 subgroups according to abscess scoring (AS-PAI 1, 2 and 3) based on the periapical index. The CP group was divided into 4 subgroups according to the periodontitis staging system (PSS1, 2,3 and 4). After recording the demographic and clinical characteristics of all participants, serum (s) and gingival crevicular fluid (GCF) samples were taken. TNF-α, IL-10, PGE2 and NO levels were measured in these samples.

**Results:**

Unlike serum measurements (sTNF-α, sIL-10, sNO and sPGE2), GCF-NO and GCF-PGE levels of the AP group were significantly higher than the control group in relation to abscess formation (54.4 ± 56.3 vs. 22.5 ± 12.6 µmol/mL, *p* < 0.001 and 100 ± 98 vs. 41 ± 28 ng/L, *p* < 0.001, respectively). Confirming this, the GCF-NO and GCF-PGE levels of the AS-PAI 1 group, in which abscesses have not yet formed, were found to be lower than those in AS-PAI 2 and 3, which are characterized by abscess formation [(16.7(3.7-117.8), 32.9(11.8-212.8) and 36.9(4.3-251.6) µmol/mL, *p* = 0,0131; 46.0(31.4–120.0), 69.6(40.3-424.2) and 74.4(32.1–471.0) ng/L, *p* = 0,0020, respectively]. Consistent with the increase in PSS, the levels of sTNF [29.8 (8.2-105.5) vs. 16.7(6.3–37.9) pg/mL, *p* < 0.001], sIL-10 [542(106–1326) vs. 190(69–411) pg/mL, *p* < 0.001], sNO [182.1(36.3–437) vs. 57.0(15.9–196) µmol/mL, *p* < 0.001], sPGE2 [344(82-1298) vs. 100(35-1178) ng/L, *p* < 0.001], GCF-NO [58.9 ± 33.6 vs. 22.5 ± 12.6 ng/L, *p* < 0.001] and GCF-PGE2 [ 99(37–365) vs. 30(13–119), *p* < 0.001] in the CP group were higher than the control group. Comparison ROC analysis revealed that the GCF-PGE2 test had the best diagnostic value for both AP and CP (sensitivity: 94.1 and 88.0; specificity: 64.0 and 78.0, respectively; *p* < 0.001).

**Conclusions:**

GCF-PE2 and GCF-NO have high diagnostic value in the determination of AP and CP, and can be selected as targets to guide treatment. In addition, the measurements of PGE2 and NO in GCF can be used as an important predictor of pulpal necrosis leading to abscess in patients with AP.

**Clinical relevance:**

In this article, it is reported that syntheses of early signaling molecules such as PGE2 and NO can be used for the diagnosis and treatment target of periapical and periodontal infections.

**Supplementary Information:**

The online version contains supplementary material available at 10.1007/s00784-024-05721-3.

## Introduction

Bone tissue has the capacity to change its structure and mass in response to mechanical effects according to mechanical requirements. However, the cellular mechanisms related to this function are not fully known. The structure of human teeth is not only specifically designed for mechanical tasks, but can also adapt to mechanical performance in order to be more efficient and maintain its role throughout life. This mechanical adaptation of the teeth is accomplished by mechanosensitive cells (such as odontoblast and cementoblast) with a biological system that senses mechanical loading or effects. A sensory extension of these cells entering into the dentinal tubule senses the signal transmission generated by external stimuli and dentinal fluid movements. Bone resorption and bone formation are modulated simultaneously by odontoblasts working as sensor cells. In general, the loading information perceived by the bones is transmitted to the effector cells, which play a role in the formation of new bone instead of the old bone, and create operational cell stress in vivo [1–3]. This stress is probably due to local hypoxia and interstitial fluid flow across the surface of odontoblasts (osteoblasts and osteocytes in other bones) and lining cells. The response of bone cells to fluid flow accelerates the expression of cyclooxygenase (COX-2), which initiates prostaglandin (PG) synthesis. In addition, endothelial nitric oxide synthase (eNOS), which mediates the adaptive response to mechanical loading in bone cells, is activated and nitric oxide (NO) production increases. In general, all of these mechanisms are similar for teeth and all bony structures of the body. It is understood here that NO and PGE2 act as early signaling molecules of the osteogenic or odontogenic response to mechanical loading or stress. These molecules also have protective and regenerative properties against reversible tooth damage [1–4]. Non-gravity or weightlessness situations where the mechanical load is too low, reduce the osteogenic response associated with NO and PG and exert a catabolic effect by affecting bone mineral metabolism.^2^ However, further research is needed on the use of signal molecules, which are thought to be involved in teeth and periodontal tissues, as diagnostic and treatment targets.

The destruction of periradicular tissues by cytokine-mediated inflammation caused by immune system cells (lymphocytes, macrophages, dendritic cells, plasma cells and polymorphonuclear leukocytes) triggered by infection is defined as apical periodontitis (AP), while chronic inflammation and destruction of periodontal tissues (alveolar bone and soft tissue) is also defined as chronic periodontitis (CP). Trauma, periodontal or endodontic infections, deterioration of the oral flora, and irritative effects caused by filling materials lie in the pathogenesis of both diseases. In addition to the harmful effects of microbial factors, when the overcoming of the host defense is added, periapical and periodontal tissue damage develops. Eventually, AP lesions in the form of reactive granulomas and cysts or loss of alveolar bone and attachment occur with bone loss [4–6]. In this context, it is thought that mechanosensitive cells [7, 8]. responsible for the production of some anabolic biological molecules such as NO, PGE2, and anti-inflammatory markers [9]. such as IL-10 may have a protective function against bone or tooth root destruction.

In this study, designed in the light of the above information, the relationship between the progression of AP and CP and the levels of TNF-α, IL-10, NO and PGE2 measured in serum and gingival crevicular fluid (GCF) samples will be investigated. In addition, these results will be compared with the results of people with healthy teeth who come for dental care and control without periodontal and periapical pathology.

## Materials and methods

### Study design

This observational, analytical, cross-sectional study was conducted at a single center. In order to reach the planned sample size, approximately 1200 patients who applied to the Department of Endodontics of Istanbul Medipol University between June 2021 and May 2022 due to apical periodontitis (AP) and/or chronic periodontitis (CP) were recorded which included their intraoral examination and demographic characteristics. As a result of the examination, 85 patients with only AP (AP group) and 50 patients with only CP (CP group), whose diagnosis was confirmed, were included in the study. Those with AP and CP at the same time or vice versa were not included in the study. AP or CP as well as aggressive periodontitis, periodontal abscess and other conditions associated with periodontal disease (those diagnosed with gingival hyperplasia and necrotizing periodontal disease) were not included in the study. Additionally, those who used steroids or nonsteroidal anti-inflammatory drugs in the last 48 h; those using high doses of biotin because it would disrupt the streptavidin-biotin interaction in the ELISA test and cause falsely high results; pregnant and/or lactating patients; people with rheumatic and collagen tissue diseases, in which the balance between bone destruction and formation is disrupted; patients with autoimmune and immunodeficiency, in which immune tolerance was impaired, as they could change the inflammatory response; patients with cancer and uncooperative patients; patients undergoing dental treatment were not included in the study. A healthy control group of 50 volunteers without periodontal pathology as well as any acute/chronic disease (muscle/joint/bone diseases, inflammatory bowel disease, local or generalized infection, severe organ disease, cardiovascular disease and diabetes mellitus) were included in the study. Those included in the research groups; randomly selected from people with similar gender, age and weight characteristics.

### Demographic-clinical history

Gender, age, body mass index (BMI), comorbidity status of all patients participating in the study were recorded. The patients’ root canal treatment (RCT), dental crown, composite/amalgam fillings (CF), number of missing teeth (NMT) findings were obtained as a result of intraoral examination.

### Periapical and periodontal disease scoring

All teeth present in the oral cavity were radiographed and the presence of radiolucent images associated with the periapical region and radiographic bone loss was assessed. Panoramic radiographs, in the study, were obtained using a digital panoramic unit (VistaPano S, Durr Dental AG, Germany), operating at 73kVp and 10 mA with 13,500 milliseconds exposure time in standard mode. For periapical radiographs Carestream RVG 5200 (RVG; Carestream Health Inc, Atlanta, CA, USA) system was used with an x-ray unit, setting of 70 kV, 8 mA. The bisecting angle technique was applied in obtaining the periapical images. Then, the radiographs were analyzed with Kodak Dental Imaging Software. From radiographic evaluation of the teeth, the presence of periapical radiolucency without periodontal disease was considered sufficient for AP diagnosis. Abscess scoring (AS-PAI) based on the periapical index was performed as an indicator of disease progression within the AP group. For this scoring, the periapical index (PAI) scoring system [10], which is a scoring system for the radiographic evaluation of apical periodontitis, was used. Accordingly, the AP group was divided into 3 subgroups. AS-PAI 1 (mild): those having at least 1 tooth with either PAI 3 or PAI 4, AS-PAI 2 (moderate): those having only 1 tooth with a PAI 5, AS-PAI 3 (severe): those having two or more teeth with a PAI 5.

Periodontal measurements, obtained for the study, consisted of the highest probing depths (in millimeters) recorded around six selected teeth per participant. In this study, the CP group was divided into 4 subgroups according to the periodontitis staging system (PSS) [12]. This system classifies the periodontitis from I to IV according to the interdental clinical attachment loss (CAL), radiographic bone loss (RBL) and tooth loss. This scoring is as follows: Stage 1: CAL 1 to 2 mm, RBL is at coronal third (< 15%) and no tooth loss, Stage 2: CAL 3 to 4 mm, RBL is at coronal third (15–33%) and no tooth loss, Stage 3: CAL > 5 mm, RBL is extending to mid-third of root and beyond and tooth loss < 4 teeth, and Stage 4: CAL > 5 mm, RBL is extending to mid-third of root and beyond and tooth loss > 5 teeth. However, Stage 1 and 2 were evaluated in the same group due to the low number of cases. As a result, the groups were designed as Stage 1–2: PSS 1–2, Stage 2: PSS 2 and Stage 3: PSS 3.

### Ethical approval

The study was approved by the Institutional Ethical Committee (Number: E-10840098-772.02-2860/344), and written consent was obtained from all participants. All patients were informed about the study and written consent was obtained from all participants.

### Collection of venous blood and gingival crevicular fluid (GCF)

Fasting (8–10 h) venous blood of all participants was taken from forearm antecubital/basic veins. After keeping the blood at room temperature for 30 min, it was centrifuged at 2500 xg for 10 min. After centrifugation, the upper serum of the tubes was separated. Hemolysis indices (HI) of the sera were measured to prevent optical interference. (Cobas 8000 Chemistry Analyzer, USA). Those with a hemolysis index greater than 50 (mg/dl Hb) were excluded from the study. Appropriate sera were stored at -80^o^C until the day of analysis. In addition to venous blood, gingival crevicular fluid (GCF) samples were also collected. GCF samples were taken prior to periodontal probing to avoid contamination by blood. To avoid contamination of the sample, patients were asked not to eat or drink anything for at least 30 min before the procedure. After selecting the area where GCF collection would be made (the area of the tooth with AP and the area that often corresponds to the same area in healthy individuals), the sampling area was isolated with cotton rolls, and plaque was removed. After gentle air-drying, PerioPaper strips (OraFlow Inc., NY, USA) were placed gently until slight resistance was felt and left there for 60 s. Three samples were taken from the mesial, distal and buccal surfaces of related tooth. Periopapers were thoroughly washed in 0.5 ml Eppendorf tubes (after subtracting the tare weight of the tube) with 100 µl of phosphate-buffered saline (PBS) using an automatic pipette. Blood-stained paper strips were removed from the samples. All GCF samples were weighed on a precision balance (Shimadzu Libror, Model AEG-220, Germany) and recorded. The samples in all closed tubes were mixed thoroughly with a vortex device (Heidolph Reax Top Vortex, Schwabach, Germany) for approximately 15–20 s. This allowed GCF to pass into PBS. Periopapers in tubes were removed from GCF and PBS without contamination. The remaining extract in tubes was stored at -80^o^C until the day of analysis. The results obtained on the study day were proportioned by weighing weights/PBS. On the day of analysis, all serums and GCF were first allowed to dissolve slowly at + 4 ^o^C and then brought to room temperature before measurement.

### Biochemical analyzes

TNF-α, IL-10, NO and PGE2 levels in the sera of all patients were measured in Microplate ELISA Reader (BioTek Epoch 2 Microplate ELISA Reader, USA) using the ELISA method. Due to the insufficient amount of sample (100 µl), only NO and PGE2 levels were measured in the GCF using the same ELISA kit.

Serum TNF-α, IL-10, NO and PGE2 levels were analyzed using ELISA plates whose wells were pre-coated with antibody (human TNF-α, IL-10, NO or PGE2). The sensitivity of TNF-α, IL-10, NO and PGE2 test kits (Bioassay Technology Laboratory, China) is 1.52 ng/L, 2.59 pg/mL, 1.12 µmol/L and 1.28 ng/L, measuring range 3-900 ng/L, respectively, 5-1500 pg/mL, 2-600 µmol/L and 2-600 ng/L, CV for all tests for intraassay and interassay were < 10%.

### Statistical analysis

Analysis of the statistical data of this study was performed using SPSS Statistic Software (25 IBM Corp, Chicago, USA). The chi-square test was used to evaluate categorical data. Whether the data showed normal distribution or not was determined using the Kolmogorov-Smirnov normality test. In comparisons consisting of more than two groups, Kruskal-Wallis Test was used in the evaluation of nonparametric data and One-way ANOVA test was used in the analysis of parametric data. The relationship between independent variables was determined by using Spearman correlations for nonparametric data and Pearson correlations for parametric data. Partial Correlation analysis was used to analyze the relationship between biochemical parameters after some parameters were controlled (after its effect is removed). Receiver operating characteristic (ROC) analysis was performed for the diagnostic performance of sPGE2, sNO, GCF-PGE2 and GCF-NO in identifying patients with AP or CP. In addition, priory power and post-hoc power analyses were performed to determine the minimum number of subjects for the study and the power of the study. Bar plots were used to graphically display nonparametric data.

### Power analysis

In the priori power analysis of independent groups (G*Power Version 3.0.10), based on the data of a study by Kumar et al. [11] comparing the PGE2 levels in GCF of patients with periodontitis and healthy individuals, it was calculated that at least 3 independent control and 3 experimental subjects were required (effect size 3.42, α = 0.05, power = 0.90). However, since the sample size must be at least 30 for parametric statistical analysis and we also want to achieve stronger statistical prediction, at least 50 subjects for the control and experimental groups were planned for this independent study.

## Results

As a result of the study, the Cohen’s d was found to be 0.74 in the effect size analysis based on the GCF-PGE2 explanatory variable of the AP and control groups. In the effect size analysis based on the GCF-PGE2 explanatory variable of the CP and control groups, Cohen’s d was found to be 1.45.

### Comparison of demographic and clinical characteristics

When the demographic characteristics of the study groups were compared, no difference was found between the groups in terms of gender, age and BMI (*p* > 0.05) (Table 1). Therefore, it was accepted that the differences between the groups in terms of other parameters were independent of these three demographic characteristics. Similarly, there was no difference between the groups in terms of number of dental crowns (0.8629). However, a statistically significant difference was found in terms of the number of RCT, CF and NMT (*p* < 0.05). The RCT and CF values of the AP group were higher than the CP group, while the NMT values of the CP group were higher than the AP group. Since attachment and tooth loss in CP and RCT and CF in AP are a general pathognomonic feature, the above findings were attributed to group characteristics. For this reason, it will not be mentioned in the discussion in order not to go beyond the subject. The mode of AS-PAI scoring in the AP group was 2 (frequency 37) and the mode of PSS scoring in the CP group was 3 (frequency 25).

**Table 1:** Comparison of demographic characteristics of groups

	AP (A)	CP (B)	Control (C)	*p* values
n	85	50	50	-
Gender, F (%)	35(41%)	25(50%)	24(48%)	^a^ 0.5575
Age, year	40 ± 11	44 ± 9	41 ± 13	^b^ 0.1075
BMI, kg/m^2^	25.2 ± 4.0	25.2 ± 2.3	24.4 ± 4.0	^b^ 0.4258
RCT, n	2.4 ± 2.0 2.0(0.0–10.0)	1.4 ± 1.8 1.0(0.0–8.0)	1.8 ± 2.4 1.0(0.0–9.0)	^c^ 0.0013
*Comparison p	< 0.01, < 0.05, > 0.05			
Dental crown, n	2 ± 4.5 0.0(0–20)	3 ± 5.9 0.0(0–23)	2 ± 3.7 0.0(0–17)	^c^ 0.8629
CF, n	5 ± 3.4 4(0–18)	2 ± 2.1 1(0–8)	3 ± 4.3 2(0–19)	^c^ <0.0001
*Comparison p	< 0.001, < 0.01, > 0.05			
NTL, n	3 ± 3.4 2(0–19)	7 ± 5.6 5(0–18)	2 ± 3.0 0,0(0–12)	^c^ <0.0001
*Comparison p	< 0.01, < 0.05, < 0.001			
AS-PAI, score	2 ± 1 2(1–3)	0	0	^−^
PSS, score	0	3 ± 1 3(1–4)	0	^−^

a Chi-square test,

b One-way ANOVA with post-test (Tukey-Kramer Multiple Comparisons Test)

c Kruskal-Wallis Test with post-test (Dunn’s Multiple Comparisons Test). *p values of between groups were compared (A-B, A-C and B-C, respectively) when p values obtained by ANOVA was < 0.05. Nonparametric data were given as mean ± SD and median (min-max) while parametric data were given as mean ± SD. SD: Standard deviation, AP: Apical periodontitis, CP: chronic periodontitis, F: Female, BMI: Body mass index, RCT: Root canal treatment, CF: Composite/Amalgam Filling, NTL: Number of tooth loss, PSS: Periodontitis Staging System (Slight: Stage 1, Moderate: Stage 2, Moderate-to-advanced (severe periodontitis with potential tooth loss): Stage 3, Advanced (severe periodontitis with potential loss of dentition): Stage 4), AS-PAI: Abscess score according to PAI in AP

### Comparison of biochemical analyses

**Fig. 1:**
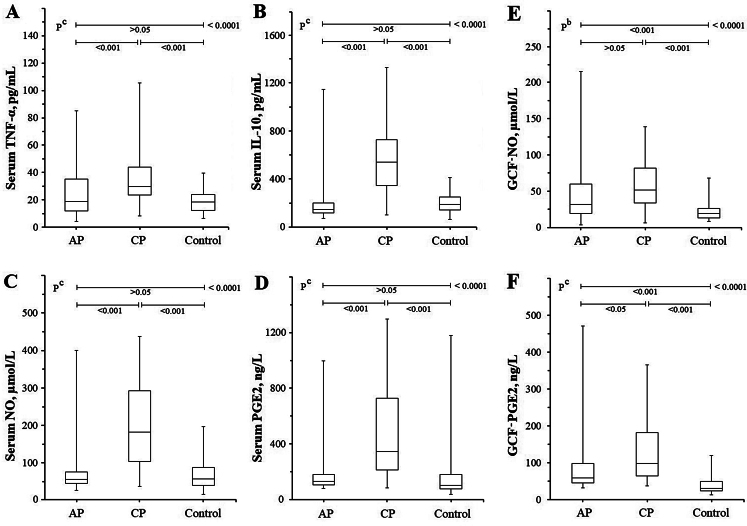
Box plot representing the sTNF-α, sIL-10, sNO, sPGE2, GCF NO and GCF PGE2 results of AP, CP and Control groups. While there was no difference between the AP and Control groups, it was observed that the (**A**) sTNF, (**B**) sIL-10, (**C**) sNO and (**d**) sPGE2 levels of the CP group were considerably higher than the other groups. (**E**) GCF NO levels of the AP and CP groups were higher than the control group. (**F**) In terms of GCF PGE2 levels, all three groups were statistically different from each other. b One-way ANOVA with post-test (Tukey-Kramer Multiple Comparisons Test), c Kruskal-Wallis Test with Post-hoc test (Dunn’s Multiple Comparisons Test)

When the groups were compared in terms of markers associated with the inflammatory process, there was no difference between the TNF-α and IL-10 levels of the AP and Control groups (*p* > 0.05), while the TNF-α and IL-10 levels of the CP group were statistically significantly higher than those of the AP and control groups (*p* < 0.01) (Table 2; Fig. 1A and B).

**Table 2:** Comparison of biochemical tests according to apical periodontitis, chronic periodontitis, and control groups

	AP (A)	CP (B)	Control (C)	*p values
n	85	50	50	-
sTNF-α, pg/mL	24.8 ± 17.5 18.7(4.3–85.3)	37.6 ± 21.3 29.8 (8.2-105.5)	17.8 ± 8.1 16.7(6.3–37.9)	^c^ <0.0001
sIL-10, pg/mL	248 ± 256 150(77-1148)	590 ± 318 542(106–1326)	204 ± 87 190(69–411)	^c^ <0.0001
sNO, µmol/mL	92.0 ± 90.9 55.7(25.8–400)	195.3 ± 108.9 182.1(36.3–437)	68.6 ± 40.2 57.0(15.9–196)	^c^ <0.0001
sPGE2, ng/L	199 ± 189 129(80–998)	484 ± 333 344(82-1298)	160 ± 175 100(35-1178)	^c^ <0.0001
GCF-NO, µmol/mL	54.4 ± 56.3	58.9 ± 33.6	22.5 ± 12.6	^b^ <0.0001
GCF-PGE2, ng/L	100 ± 98 59(31–471)	128 ± 80 99(37–365)	41 ± 28 30(13–119)	^c^ <0.0001

b One-way ANOVA with post-test (Tukey-Kramer Multiple Comparisons Test),

c Kruskal-Wallis Test with Post-hoc test (Dunn’s Multiple Comparisons Test). *If the P value is significant (< 0.05) as a result of the ANOVA test, the P values between the groups are compared (A-B, A-C and B-C, respectively) (Fig. 1). Nonparametric data were given as mean ± standard deviation and median (min-max). AP: Apical periodontitis, CP: chronic periodontitis, sTNF-α: Serum tumor necrosis factor-alpha, sIL-10: Serum interleukin-10, sNO: Serum nitric oxide, sPGE2: Serum prostaglandin E2, GCF: Gingival crevicular fluid

When the groups were evaluated in terms of NO and PGE2, which are thought to be protective against inflammatory or mechanical stresses of the tooth, the difference between sNO and sPGE2 levels between the AP and Control groups was not statistically significant (*p* > 0.05). The elevation in sNO and sPGE2 levels of the CP group compared to the AP and control groups was statistically significant (*p* < 0.01) (Table 2; Fig. 1C and D). When the groups were compared in terms of the levels of these signal molecules in GCF, there was no statistically difference between the GCF-NO levels of the AP and CP groups (*p* > 0.05) (Fig. 1E), while the GCF-NO levels of both groups were found to be statistically higher than the control group (*p* < 0.01). Interestingly, all three groups were statistically different from each other, with GCF-PGE2 levels ranked as CP > AP > Control group (*p* < 0.01) (Fig. 1F).

**Fig. 2:**
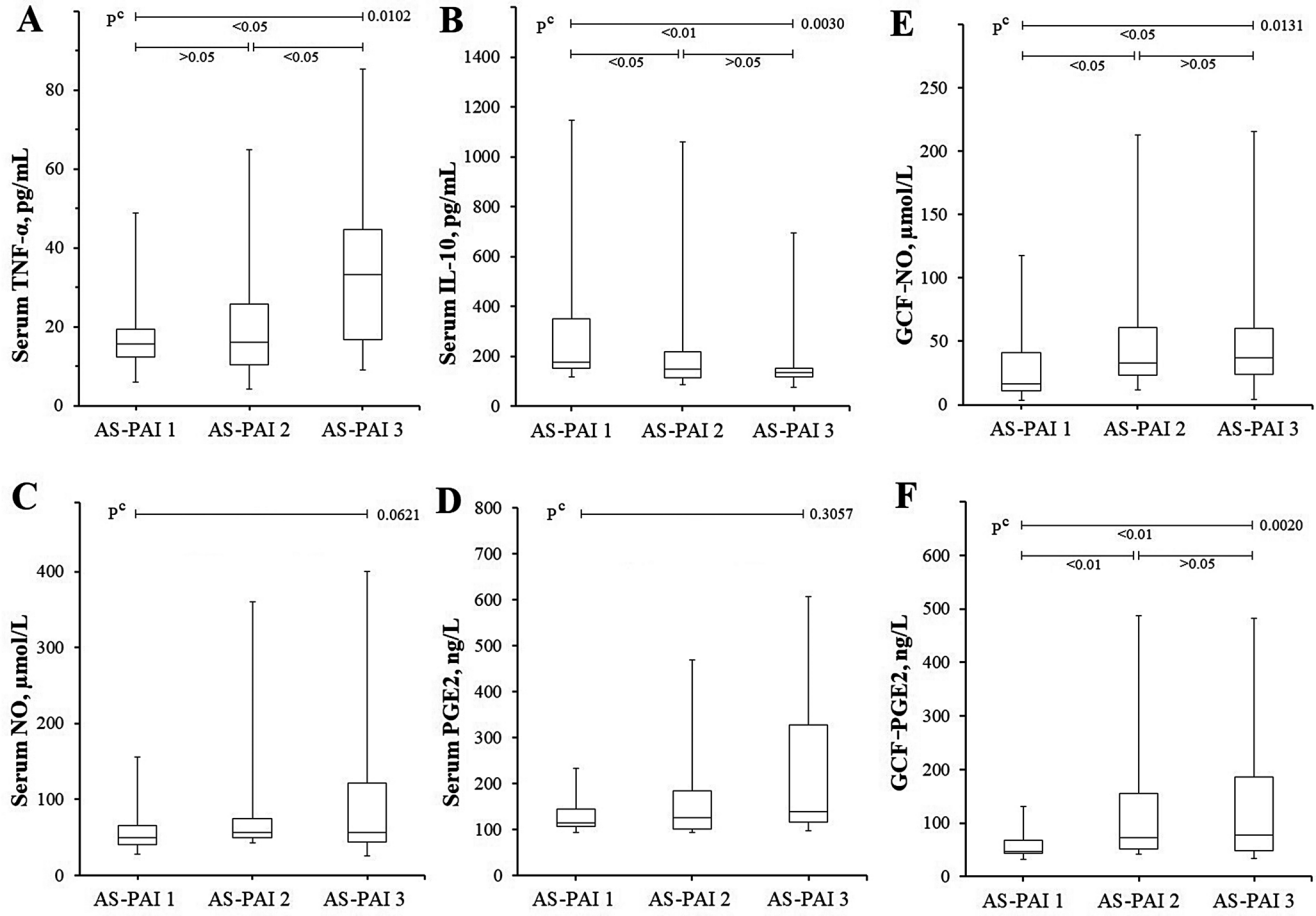
Box plot representing the sTNF-α, sIL-10, sNO, sPGE2, GCF NO and GCF PGE2 results of AP group according to AS-PAI score. (**A**) While there is no difference between AS-PAI 1 and AS-PAI 2 in terms of TNF-α, it is seen that the TNF-α levels of AS-PAI 3 are higher than both subgroups. (**B**) While there is no difference between AS-PAI 2 and AS-PAI 3 in terms of sIL-10, it is seen that the sIL-10 levels of AS-PAI 1 are higher than both subgroups. (**C**) The sNO and (**D**) sPGE2 levels did not differ between subgroups. (**E**) The GCf NO and (**F**) GCF PGE2 levels seem to be higher in AS-PAI 2 and AS-PAI 3 subgroups compared to AS-PAI 1. c Kruskal-Wallis Test with Post-hoc test (Dunn’s Multiple Comparisons Test)

When patients with AP were subgrouped radiologically according to the severity of the disease (Table 3), 44% of the cases were in the AS-PAI 2 subgroup, in which the lesion was the first to appear. When subgroups with AP were compared in terms of serum TNF-α, which is an important indicator of tissue destruction and inflammation, there was no difference between AS-PAI 1 and AS-PAI 2 subgroups (*p* > 0.05), while TNF-α levels of the AS-PAI 3 subgroup were higher than the others (*p* < 0.05) (Fig. 2A). In contrast to TNF-α levels, IL-10 levels were higher in the AS-PAI 1 subgroup than in the others (*p* < 0.05). The difference between AS-PAI 2 and AS-PAI 3 was statistically insignificant (*p* > 0.05) (Fig. 2B). Despite no statistically significant differences between AS-PAI subgroups in sNO and sPGE2 (Fig. 2C and 2D), it is a remarkable finding that GCF-NO and GCF-PGE2 levels in both AS-PAI 2 and AS-PAI 3 were significantly higher than AS-PAI 1 (Fig. 2E F).

**Table 3:** Comparison of TNF-, IL-10, NO and PGE2 results of patients with AP according to abscess score

	AS-PAI 1 (A)	AS-PAI 2 (B)	AS-PAI 3 (C)	*P values
n	21	37	27	-
sTNF-α, pg/mL	17.5 ± 9.2 15.7(5.9–48.8)	22.6 ± 16.8 16.0(4.3–64.9)	33.5 ± 20.1 33.2(9.2–85.3)	^c^0.0102
sIL-10, pg/mL	367 ± 355 177(117–1148)	244 ± 243 149(87-1061)	160 ± 114 134(77–695)	^c^0.0030
sNO, µmol/L	57.9 ± 31.0 49.1(27.8-155.5)	95.9 ± 91.7 56.3(42.6-360.5)	113.1 ± 113.5 56.6(25.8-400.4)	^c^0.0621
sPGE2, ng/L	136 ± 50 115(94–300)	197 ± 203 126(88–998)	250 ± 226 139(80–893)	^c^0.3057
GCF-NO, µmol/mL	30.2 ± 28.3 16.7(3.7-117.8)	61.5 ± 61.0 32.9(11.8-212.8)	66.3 ± 68.7 36.9(4.3-251.6)	^c^0.0130
GCF-PGE2, ng/L	52.1 ± 20.7 46.0(31.4–120.0)	105.3 ± 100.1 69.6(40.3-424.2)	130.2 ± 117.2 74.4(32.1–471.0)	^c^0.0020

c Kruskal-Wallis nonparametric ANOVA Test with Post-hoc test (Dunn’s Multiple Comparisons Test). * If the P value is significant (< 0.05) as a result of the ANOVA test, the P values between the groups are compared (A-B, A-C and B-C, respectively) (Fig. 3). Nonparametric data were given as median (min-max). sTNF-α: Serum tumor necrosis factor-alpha, sIL-10: Serum interleukin-10, sNO: Serum nitric oxide, sPGE2: Serum prostaglandin E2, GCF: Gingival crevicular fluid, AS-PAI: Abscess score based on periapical index (PAI)

When the subgroups were analyzed according to the progression of the pathology in CP (Table 4), the sTNF-α and sNO levels of the PSS 4 subgroup were found to be higher than the PSS 1–2 and PSS 3 subgroups (*p* < 0.01) (Fig. 3A and 3 C). While there was no difference in sPGE2 levels in PSS subgroups, the increase observed in sIL-10 levels was found to be statistically significant (*p* < 0.05), which was consistent with the progression in PSS, unlike AS-PAI scoring (Fig. 2B and 2D). While there was no difference between the other subgroups, the GCF-NO and GCF-PGE2 levels of PSS 4 were significantly higher than the PSS 1–2 subgroup (*p* < 0.01).

**Table 4:** Comparison of TNF-, IL-10, NO and PGE2 results of patients with chronic periodontitis according to PSS

	PSS 1–2 (A)	PSS 3 (B)	PSS 4 (C)	*P values
n	10	25	15	-
sTNF-α, pg/mL	26.0 ± 11.6 25.7(8.2–52.3)	31.2 ± 13.2 29.1(13.0-66.1)	56.1 ± 25.8 45.2(23.4-105.5)	^c^0.0006
sIL-10, pg/mL	243 ± 90 251(106–366)	548 ± 211 534(137–1211)	893 ± 292 968(298–1326)	^c^< 0.0001
sNO, µmol/L	107 ± 43 105(43–193)	171 ± 84 177(36–343)	295 ± 104 318(79–437)	^c^< 0.0001
sPGE2, ng/L	371 ± 305 240(134–1044)	450 ± 322 317(82-1298)	615 ± 346 499(181–1291)	^c^ 0.1084
GCF-NO, µmol/mL	33.5 ± 17.6 30.3(6.9–67.5)	55.5 ± 30.3 52.2(6.4-118.9)	81.7 ± 33.7 83.2(29.5-138.9)	^c^ 0.0016
GCF-PGE2, ng/L	79 ± 47 67(37–185)	119 ± 65 95(40–267)	177 ± 97 146(55–365)	0.0088

c Kruskal-Wallis Test (nonparametric ANOVA) with Post-hoc test (Dunn’s Multiple Comparisons Test). *When P value obtained by ANOVA was < 0.05 (significant), P values of between groups were compared (A-B, A-C and B-C, respectively). Nonparametric data were given as median (min-max). sTNF-α: Serum tumor necrosis factor-alpha, sIL-10: Serum interleukin-10, sNO: Serum nitric oxide, sPGE2: Serum prostaglandin E2, GCF: Gingival crevicular fluid, PSS: Periodontitis staging system (due to the insufficient number of PSS 1 and PSS 2 samples, both were included in the same group and evaluated)

**Fig. 3:**
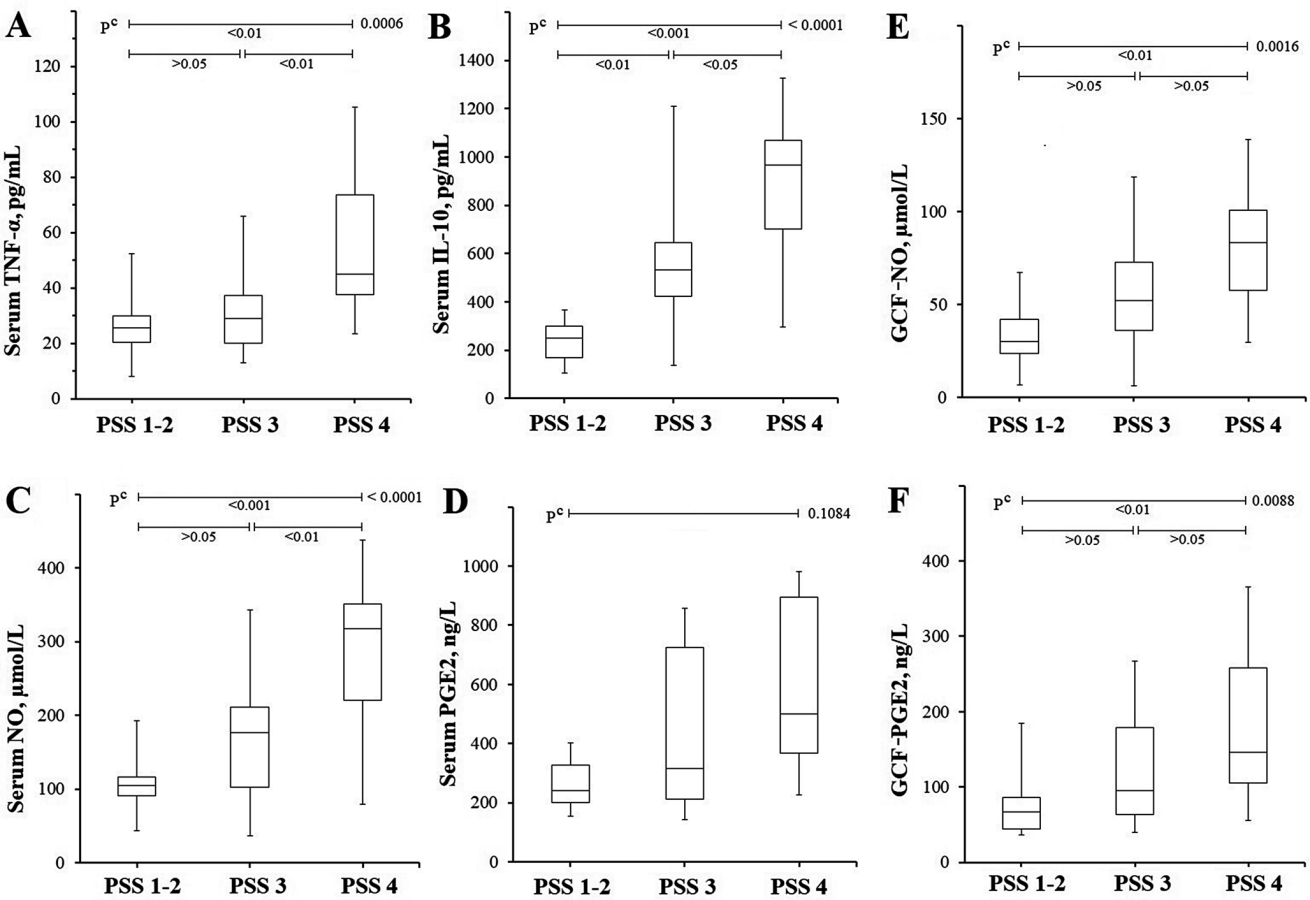
Box plot representing the sTNF-α, sIL-10, sNO, sPGE2, GCF NO and GCF PGE2 results of CP group according to PSS score. (**A**) While there is no difference between PSS 1–2 and PSS 3 in terms of sTNF-α, it is seen that the sTNF-α levels of PSS 4 are higher than both subgroups. (**B**) It is seen that sIL-10 levels increase in parallel with the progression of PSS, with a statistically significant difference between PSS subgroups. (**C**) It is seen that sNO levels of PSS 4 subgroup are higher than other subgroups. (**D**) The sPGE2 levels did not differ between subgroups. (**E**) The GCF NO and (**F**) GCF PGE2 levels were higher in the PSS 4 than the PSS 1–2. c Kruskal-Wallis Test with Post-hoc test (Dunn’s Multiple Comparisons Test)

**Fig. 4:**
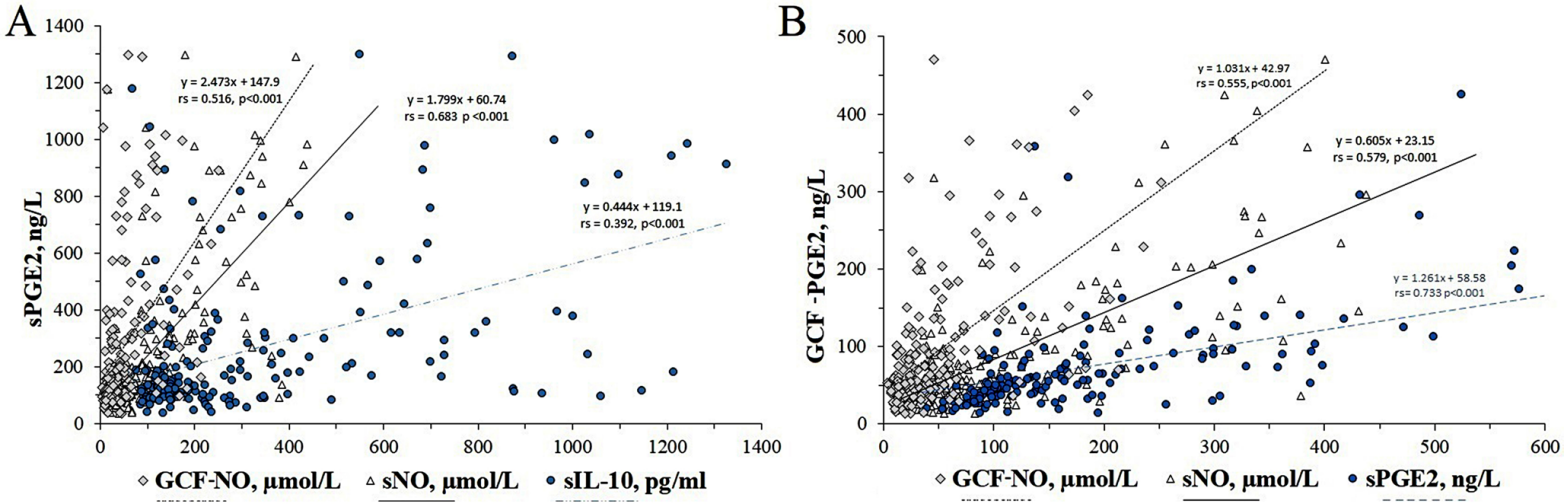
Scatter plot representing sIL-10, sNO, sPGE2 and GCF NO values generated against the sPGE2 and GCF PGE2 variables. (**A**) The figure shows a statistically significant correlation between sPGE2 and sIL-10, sNO, and GCF NO ranging from mild to moderate. (**B**) The figure shows a statistically significant correlation between GCF PGE2 and sNO, sPGE2, and GCF NO ranging from mild to moderate. rs: Spearman correlation coefficient

In the correlation matrix analysis based on all participants, a positive low to moderate correlation was found between sPGE2 and sIL-10, sNO, GCF-NO and GCF-PGE2 levels, respectively (Table 5). However, when the analyzes of the data of these variables are repeated with appropriate methods according to whether they are parametric or not; values close to the correlation matrix results were obtained between sPGE2 levels and sIL-10 (rs = 0.3924, 95% CI: 0.259 to 0.511, *p* < 0.0001), sNO (rs = 0.6828, 95% CI: 0.595 to 0.756, *p* < 0.0001), GCF-NO (rs = 0.5163, 95% CI: 0.398 to 0.617, *p* < 0.0001) and GCF-PGE2 (rs = 0.7329, 95% CI: 0.656 to 0.790, *p* < 0.0001) levels (Fig. 4). Similarly, there was a positive correlation between sNO and GCF NO (rs = 0.6757, 95% CI: 0.586 to 0.749, *p* < 0.0001) and between GCF NO and GCF PGE2 (rs = 0.5555, 95% CI: 0.444 to 0.650, *p* < 0.0001). Correlations between sPGE2 and sTNF-α, GCF PGE2 with sIL-10, sTNF-α with GCF-NO and sTNF-α were < 0.30.

**Table 5:** Correlation matrix results

*n*: 185	A:	B:	C:	D:	E:	F:
A: PGE2	1.000					
B: TNF-α	0.2966	1.000				
C: IL-10	0.4815	0.2961	1.0000			
D: NO	0.6686	0.3503	0.6003	1.0000		
E: GCF-NO	0.4279	0.2518	0.3130	0.6191	1.0000	
F: GCF-PGE2	0.6892	0.2682	0.3698	0.7028	0.5580	1.000

Correlation matrix analysis: According to whether the data are parametric or not, the evaluation is made again with Pearson (r) or Spearman (rs) correlation analysis. Correlations of ≥0.30 were considered to be significant in this study

When the effect of the inflammatory and anti-inflammatory markers TNF-α and IL-10 was eliminated (controlled) by partial correlation analysis, the net correlations between sPGE2 and sNO and between GCF-PGE2 and GCF-NO were found to be r_partial_: 0.522 and 0.485, respectively (R^2^: 0.272 and 0.235, respectively, *p* < 0.0001). In other words, it was determined that 27.2% of the total change (variance) in sPGE was caused by sNO and 23.5% of the total change in GCF-PGE2 was due to GCF-NO.

**Fig. 5:**
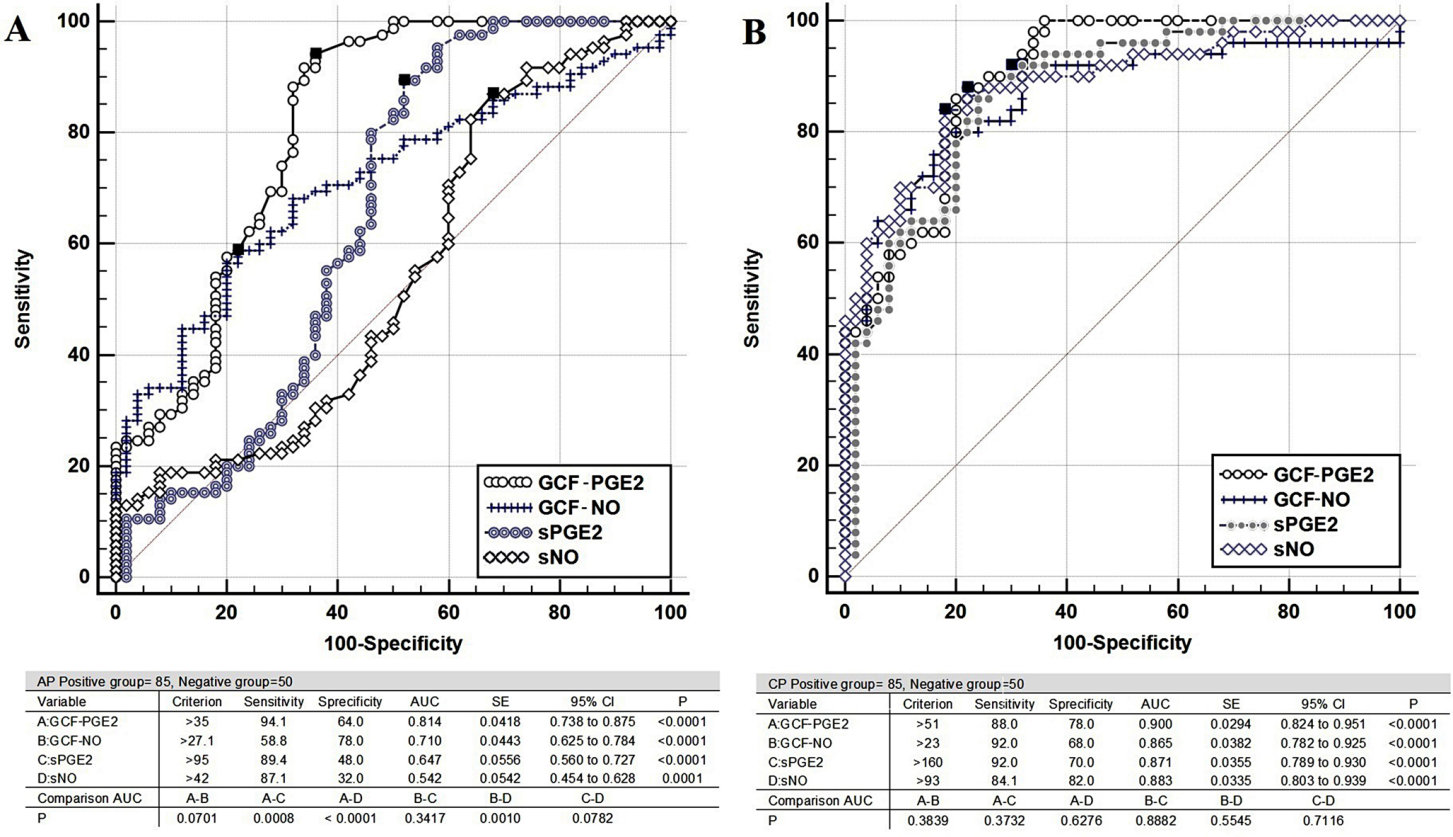
Comparison of receiver operating characteristic (ROC) analysis results and graph used to compare the diagnostic power of sPGE2, sNO, GCF-PGE2 and GCF-NO parameters to determine (**A**) AP and (**B**) CP. The highest diagnostic power belongs to the GCF-PGE2 independent variable

In the comparison ROC analysis (Fig. 5) performed to evaluate the diagnostic power (performance) of the tests to be used to detect AP and to determine the best test, GCF-PGE2 was found to be the best test (Fig. 5). The best cut-off value of the test was found to be > 35 ng/L, sensitivity 94.1%, specificity 64.0%, and AUC (Area Under the Curve) 0.814 (*p* < 0.0001). In second place was the GCF-NO test, which was also measured in the GCF, and the best threshold value of the test was > 27.1 µmol/L, the sensitivity was 58.8%, the specificity was 78.0%, and the AUC was 0.710 (*p* < 0.0001). The diagnostic value of the GCF-PGE2 test was statistically significantly higher than sPGE2 and sNO (*p* < 0.001).

Odds ratio was calculated by Fisher’s Exact Test based on the criteria determined for GCF-PGE2 in the ROC analysis (cut-off for AP: >35 ng/L and for CP: >51 ng/L). It was found that the probability of GCF-PGE2 value > 35 ng/L in the AP group was 22.095 (95% CI: 7.624 to 64.036, *p* < 0.0001) times more than the control group. The probability of GCF-PGE2 value > 51 ng/L in the CP group was 22.095 (95% CI: 8.791 to 76.897, *p* < 0.0001) times more than the control group, too.

## Discussion

Loss of the fight between bacterial attack and host defense, which is responsible for periodontal and endodontic disease pathogenicity, against the host may cause permanent damage to the periapical tissue and periodontium, which may lead to tooth loss. Pathogenic bacteria or their toxic products can progress to the periapex of the root in a tightly protected environment. Localized inflammatory and protective processes occurring in the pulp against the damage that begins to develop here, may result in the formation of a reactive (tertiary) dentin layer or necrosis with reduced fluid permeability, which is relatively less in the number of tubules and continuity between the tubules [12–16]. The host’s dynamic response, which consists of cellular and humoral immune cells, cannot reach the root canal system when this condition occurs and therefore endodontic treatment is often required.^15^ Therefore, accurate and early diagnosis of both AP and CP, which share similar biological pathways, will increase the likelihood of treatment. For this purpose, the use of inflammation-related markers such as TNF-α, IL-1β, IL-6 and IL-10 [17], and biomarkers with protective functions such as PGE2 and NO^2^ released from mechanosensitive cells, have come to the fore in the diagnostic field. In addition, it is known that some molecules such as Tissue Growth factor-beta (TGF-β) and prostaglandin, which suppress odontoclastic activity and stimulate regeneration-related odontoblastic activity, and activated neutrophils and T cells, are released in chronic periodontal infections [14]. In this study, the high levels of NO and PGE2 signaling molecules, especially in GCF samples of patients with AP and CP, compared to the control group, were attributed to their increased expression due to their protective functions in response to damage. Again, the increase in PGE2 levels observed in GCF samples was more pronounced in CP patients compared to the control and AP groups, respectively, indicating that it can be used in the diagnostic field in relation to the extent of tissue damage.

The NO and prostaglandin synthesis pathways associated with infections share many similar mechanisms that enable them to be produced simultaneously in the same tissues. However, the mechanisms that positively or negatively regulate the prostaglandin production of NO and the interaction between these two are still unclear [18, 19]. In this study, which we conducted in the context of understanding AP and CP and creating new treatment targets, the moderate correlation between PGE2 and NO, which was determined by the analysis with partial correlation, purified with other variables, provides explanatory answers to these discussions. The pathophysiological basis of this determined relationship is based on periodontal inflammation and bacterial infections that predispose to damage [20, 21]. Bacteria secrete products (remnants such as lipopolysaccharides, proteoglycans, and toxins) that can trigger inflammation and tissue destruction. These foreign products are potent activators for host defense cells. This activation causes overexpression of TNF α, IL-1, IL-6, NO and PGE2 levels [22–24]. This means that inflammatory conditions can increase the formation of reactive oxygen species (ROS), which disrupt the redox balance in the body and cause oxidative damage. Therefore, triggered oxidative damage can damage many tissues, including protein and lipid oxidation as well as DNA damage [25]. In recent years, evidence has been presented that oxidative stress may be more than a simple consequence of inflammation and that some antioxidants that lower oxidative stress markers may provide additional therapeutic support, although not yet conclusively proven, in cases where standard treatment of periodontitis has failed [26]. Gomes and colleagues [27], who also conducted a study examining the relationship between oxidative stress and AP, reported that there is a relationship between increased root canal endotoxin levels, chronic apical periodontitis, increased oxidative and nitrosative stress, severity of depression and decreased quality of life. Additionally, a positive relationship was found between root canal lipopolysaccharide levels (endotoxin), chronic AP, depression severity and oxidative and nitrosative stress markers, NO and total radical trapping antioxidant parameter (TRAP). In this study we conducted, the finding of a relationship between abscess severity calculated based on PAI and GCF-NO in patients with AP confirms the above information.

The correlations observed between PGE, NO, and IL-10 in this study may also be attributed to their simultaneous expression and inhibition by the same pathways (STAT-1 and PI3K/Akt). The acute phase response with these mediators (TNF α, IL-1, IL-6, NO and PGE2) creates local or systemic effects as a result of triggering catabolic and anabolic mechanisms in bone or soft tissue [28–30]. Catabolic or anabolic dominance is determined by the balance between the extent of the infection and the host response to it. Some researchers’ finding that inflammation induces NO production from inducible nitric oxide synthase (iNOS), H2O2 production from NADPH and xanthine oxidase, and prostaglandin production from Cyclooxygenase (COX) [31, 32] supports our finding that NO increased in this study, especially due to chronic periodontitis.

In the initial stages of apical periodontitis, which can occur due to bacterial invasion, trauma, and chemical irritation, there are throbbing pain, tenderness with pressure, and pain on chewing. At this stage, the inflammation is limited to the periodontal ligament and radiographic changes are indistinguishable since there is no significant tissue damage [14]. This stage coincides with the AS-PAI 1 stage of AP in our study. In our study, we reached a biomarker that can distinguish this stage (AS-PAI 1) from severe periodontitis with exacerbating features (AS-PAI 2 and 3 stages). The fact that both GCF-PGE2 and GCF-NO levels were lower in AS-PAI 1 compared to the others at the end of the study showed that these two biomarkers could be used as evidence of severe periodontitis’ exacerbating features. This finding can be explained as follows. The vascular system of the pulp is responsible for supplying the tissue with oxygen, water, nutrients and immune cells, while removing bacterial byproducts and waste. This vascular network of the pulp actively aids in the inflammatory response (transmission of reactive molecules with the help of capillary network) of the pulp against infections and also its regeneration [33, 34]. When the pulp infection progresses and reaches the pulpal avascularity state, the cells or tissues in the pulp go into necrosis and an abscess is formed. Pulp necrosis also removes the apical neurovascular structure and the fluid permeable tubular dentin layer [16, 35, 36]. Thus, molecules such as PGE2 and NO, which cannot possibly be transported by blood or fluid circulation, pass from the interstitial space in the tissue to the gingival sulcus, causing their high detection. This is the answer to why we find high PGE2 and NO in AS-PAI 2 and 3, which is characterized by severe exacerbating features. Moreover, the fact that we determined the diagnostic value of GCF-PGE2 and GCF-NO test to be higher in ROC analysis compared to serum is the proof of our hypothesis. An investigation by Belmar et al. [37] to monitor chronic apical periodontitis found high levels of MMP-9 and MMP-2 in gingival crevicular fluid of patients, confirming our hypothesis. Another possible reason for the high detection of GCF-PGE2 and GCF-NO in AS-PAI 2 and 3 is that NO, which is a highly reactive radical, is involved in immune system regulation, vasodilator function, COX activation, and as well as protection against infections like some prostaglandins [31, 38]. All this information and our research findings are evidence that NO can be selected as a treatment target.

While there was no difference between the subgroups of AP in terms of the levels measured in serum, the high levels of GCF-NO and GCF-PGE2 in AS-PAI 2 and AS-PAI 3 indicate that some molecules related to the formation or destruction in periodontitis, which is a very local pathology, are not sufficiently introduced into the bloodstream. It was seen as evidence that GCF would provide more useful information than systemic blood circulation in understanding periodontal and periapical pathologies. In addition, these results are consistent with the knowledge that NO and PGE2 are involved in the transmission of mechanical stress that creates fluid shear stress and stimulate bone remodeling [39–43]. NO achieves this task by activating protein kinase G via cGMP and exhibiting anti-inflammatory behavior. This pathway is also associated with proliferation, differentiation and cell survival of bone homeostasis. In a systematic analysis of the regulatory role of NO, Yan et al. [41] concluded that NO affects bone metabolism by showing complex effects on osteogenesis and osteoclastogenesis by mediating mechanical signals.

TNF-α, a proinflammatory agent activated by bacterial products (such as lipopolysaccharide) and regulated by the IkB/NF-kB transduction pathway, is responsible for the initiation of inflammatory events. In contrast, IL-10, which suppresses advanced inflammation, provides control of damage [38]. In our study, the higher sTNF-α and sIL-10 levels of the CP group, unlike AP, were associated with a better reflection of the reactive state due to infection in the blood circulation in patients with CP. The available data suggested that the changes in these markers may be more prominently reflected in the blood only in the AS-PAI 3 subgroup, where the pathology is fully evident. Contrary to the increase in TNF-α, the decrease observed in IL-10 is an indication of a better response to limit the pathology in the AS-PAI 1 subgroup due to the protective role of IL-10 [9, 38]. In addition, low sIL-10 levels in AS-PAI 2 and 3 subgroups suggest that bone loss is experienced in these patients due to insufficient elevation of IL-10. Therefore, stimulation of IL-10, like eNOS, can be targeted to guide treatment.

The increase in sTNF-α and sNO levels in the PSS 4 subgroup, where the pathology in CP is the most advanced, was attributed to the fact that the inflammation was more severe in this patient group and to the stimulation of NO synthesis due to the activation of the iNOS pathway by proinflammatory cytokines such as TNF-α. It was thought that this might be related to the progression of attachment and bone loss observed in CP [5, 38]. Van’t Hof and Ralston described an inverse mechanism for the action of NO in a systemic review they wrote [44]. They reported that unlike iNOS, NO derived from the eNOS pathway can act together with prostaglandins, promoting bone formation and inhibiting bone resorption. Osteoclasts (odontoblasts in teeth), one of the two main cell types responsible for bone remodeling, derive from the monocyte-macrophage lineage and are responsible for bone resorption and apoptosis. Osteoblasts with tubular extensions (odontoblasts in teeth) are cells of mesenchymal origin. They act as mechanical stress sensors and are capable of forming new bone matrix [45, 46]. Both these cell types are affected by NO, which is regulated by local factors such as prostaglandins, growth factors, and cytokines, but they function differently. Similar to this physiopathological paradox is the increase we observed in sIL-10 levels, which is consistent with the progression in PSS as opposed to AS-PAI scoring. This finding is evidence that IL-10 functions in a different direction in AP, which is a more local pathology compared to CP. In contrast to the protective role of IL-10 in AP, which triggers the regeneration of the bony tissue and is characterized by an increase in tissue destruction in its insufficiency, in CP, which is a pathology predominantly associated with the soft tissue surrounding the teeth (gingival tissues and periodontal ligament), there is an increase in parallel with inflammation to limit inflammation. Zang and Won also found information confirming our findings [9, 38]. In addition, the high levels of GCF-NO and GCF-PGE2 that we observed in the PSS 4 subgroup of CP also reinforce the above explanation.

In this study, the higher diagnostic value of PGE2 and NO measured in GCF than serum results was attributed to the fact that AP is a highly localized infection and the regional production of inflammatory cytokines, PGE and NO triggered by the defense response against pathogens [35, 47]. Although immune cells and molecules come into play against severe infection that endanger pulp vitality by causing cell damage, host defense is insufficient in removing pathogens because the only exit of the tooth is the narrow apical foramen and these cells cannot sufficiently penetrate the hard layer of dentin [35]. Therefore, the pathology is more localized and its reflection on the systemic circulation is limited. In addition, the high probability of having higher GCF-PGE2 levels in AP and CP compared to healthy patients confirms the findings.

Periodontitis, which affects millions of people worldwide and is the most common cause of tooth loss, has been found to be a risk factor for many chronic diseases, including cardiovascular, rheumatic and degenerative diseases [48–50]. Therefore, the pathogenesis of effective fight against periodontitis needs to be well known. Plaque biofilm [51], epithelial and immune cells, odontoclasts, odontoblasts, fibroblasts, inflammatory cytokines, tissue destructive molecules [4–6, 14] and recently discovered nucleotide-binding oligomerization domain-like receptor (NLR) complexes called inflammasome [52, 53] play a very complex role in the pathogenesis of periodontitis. Many molecules involved in pathogenesis have been investigated for years. Among these, the most studied in recent years are inflammasomes and their negative regulators, which play important roles in the formation of molecular infections, as they are the basic regulators of the innate immune system [52, 54]. However, unfortunately, molecules that can be used definitively in the diagnosis of periodontitis have not yet been identified. Therefore, in this study, we studied NO and PGE2, which we think play a role in the negative regulation of inflammation, as a potential therapeutic target in the treatment of periapical and periodontitis diseases. In our study, the possible reason for the increase in serum and GCF NO and PGE2 levels consistent with the severity of AP and CP may be the effort of cells to limit the inflammatory molecules (such as inflammasomes) that play an important role in the formation of periapical and periodontitis diseases by increasing NO and PGE2 production. Moreover, a study conducted by Sokolowska et al. [54] in human macrophages to observe the effect of PGE2 on inflammasomes supports our conclusion. These researchers demonstrated that NLR Family Pyrin Domain Containing 3 (NLRP3)-mediated inflammasome activation in human primary monocyte-derived macrophages was inhibited by PGE2. They found that PGE2 does this by increasing intracellular cAMP. They also observed that adenylate cyclase inhibitors reversed PGE2-mediated NLRP3 inhibition. All of these provide evidence for the molecular explanation of our study results.

In this study, the significant increase in the GCF-PGE2 and GCF-NO levels of the PSS 4 subgroup suggested its use in patients with CP as an indicator of advanced periodontal stages. This finding, on the one hand, agrees with the knowledge of Orkun et al. that NO detected in GCF is a good early detection marker of periodontal inflammation [55], on the other hand, it contradicts their findings that GCF-NO does not show a significant difference between the onset and advanced stages of periodontal disease. The possible reason for this discrepancy was attributed to the fact that these researchers worked with a small number of patients and did not make scoring according to PSS. In a study of 30 patients with chronic periodontitis, Hussein et al. [56] found a strong correlation between PGE2 measured in GCF and severity of chronic periodontitis and this finding agrees with ours. All these outputs indicate that GCF fluid will also provide useful information in PSS staging of CP.

One of the limitations of this study is the inability to measure IL-10 and TNF-α values in GCF fluid due to low sample volume, they were only measured in circulation.

## Conclusion

GCF-PGE2 and GCF-NO have high diagnostic value in the determination of AP and CP and can be selected as targets to guide therapy. Measurements of PGE2 and NO in GCF can be used as an important predictor of pulpal necrosis leading to severe periapical destruction in patients with AP. In addition, GCF-NO levels are associated with GCF-PGE2 levels in periodontal infections.

### Electronic supplementary material

Below is the link to the electronic supplementary material.

Supplementary Material 1

## Data Availability

Data used or analyzed in this investigation are included within the manuscript or available upon reasonable request from the corresponding author.
